# Progress of Dispersants for Coal Water Slurry

**DOI:** 10.3390/molecules28237683

**Published:** 2023-11-21

**Authors:** Xiaotian Liu, Shan Wang, Ning Liu, Bo Wei, Tian An

**Affiliations:** Key Laboratory of Coal Clean Conversion & Chemical Engineering Process, Xinjiang Uygur Autonomous Region, School of Chemical Engineering, Xinjiang University, Urumqi 830046, China; 107552103542@stu.xju.edu.cn (X.L.); wangshanw0628@xju.edu.cn (S.W.); nlln170@126.com (N.L.); 107552201259@stu.xju.edu.cn (T.A.)

**Keywords:** coal water slurry dispersant, dispersant type, three-dimensional structure dispersant, adsorption performance, dispersion mechanism

## Abstract

Dispersants, serving as an essential raw material in the formulation of coal water slurry, offer an economical and convenient solution for enhancing slurry concentration, thus stimulating significant interest in the development of novel and efficient dispersants. This paper intends to illuminate the evolution of dispersants by examining both the traditional and the newly conceived types and elaborating on their respective mechanisms of influence on slurry performance. Dispersants can be classified into anionic, cationic, amphoteric, and non-ionic types based on their dissociation properties. They can be produced by modifying either natural or synthetic products. The molecular structure of a dispersant allows for further categorization into one-dimensional, two-dimensional, or three-dimensional structure dispersants. This document succinctly outlines dispersants derived from natural products, three-dimensional structure dispersants, common anionic dispersants such as lignin and naphthalene, and amphoteric and non-ionic dispersants. Subsequently, the adsorption mechanism of dispersants, governed by either electrostatic attraction or functional group effects, is elucidated. The three mechanisms through which dispersants alter the surface properties of coal, namely the wetting dispersion effect, electrostatic repulsion effect, and steric hindrance effect, are also explained. The paper concludes with an exploration of the challenges and emerging trends in the domain of dispersants.

## 1. Introduction

Coal serves a pivotal role in the energy frameworks of countries like China and India [1,2]. Considering the energy development trajectory in these nations, the dominance of coal as a primary energy source is unlikely to alter significantly [3]. Notably, coal is a non-renewable energy resource. The judicious extraction and consumption of coal resources exemplify effective strategies to conserve energy and safeguard the ecological environment. Such an approach carries immense practical relevance for achieving sustainable development within the coal industry. Moreover, in light of low oil reserves and energy shortcomings in numerous countries, the advancement of coal-based alternative fuels and exploration for viable oil substitutes is deemed crucial [4]. Coal water slurry (CWS) is considered a promising oil substitute. It is a coal-based, fluid clean fuel, characterized by low pollution levels, economical cost, and superior combustion efficiency [5,6].

A typical CWS primarily comprises 60–75% coal, 25–40% water, and approximately 1% additives [7]. Despite exhibiting high viscosity, CWS is susceptible to stratification and instability due to the coal surface’s hydrophobic nature [8]. Dispersants, the most critical additive in CWS preparation, are amphoteric substances with both hydrophilic and hydrophobic groups. They enhance adsorption on the potential coal surface, enabling uniform dispersion of coal particles in water. This consequently decreases the viscosity of CWS, thereby improving its flowability [9].

The efficacy of CWS is primarily influenced by the quality of the coal, dispersants, and the gradation of the particle size. Among these, dispersants, alongside coal quality, are perceived as the most crucial factors constraining CWS performance [10]. In recent times, low-rank coals, primarily lignite, non-sticky coal, and long-flame coal, known for their subpar slurry properties, have been extensively employed in CWS preparation. This necessitates the chosen dispersants to exhibit superior viscosity reduction and slurry stabilization properties [11,12,13]. Nevertheless, the diverseness of coal properties and the preferential use of industrial wastewater for pulping in practical applications considerably restrict the universality of dispersants. This necessitates the design of specific dispersants for diverse coal types [14]. The development of new dispersants has revealed that their structure significantly influences their performance. Hence, targeted structure design is pivotal to augment the efficacy of dispersants. Moreover, in industrial contexts, the utilization of low-cost and eco-friendly raw materials is a vital strategy to curtail dispersant costs. This paper supplements existing classification methods of dispersants from structural and raw material source perspectives and elaborates on their mechanisms in detail.

## 2. Classification and Characteristics of CWS Dispersants

Dispersants denote the surfactants added to CWS that enable stable dispersion of coal particles in water, preventing stratification and precipitation over extended periods [15]. As significant additives in CWS preparation, dispersants can adhere to the coal surface, altering its properties and thereby enhancing CWS performance [16].

We provide a comprehensive summarization of coal water slurry dispersants [17]. Diverse types of CWS dispersants exist, as illustrated in Table 1. Currently, based on their dissociation attributes, they are categorized into non-ionic and ionic dispersants [18]. Non-ionic dispersants chiefly comprise polyoxyethylene ether, non-ionic aliphatic compounds, and some natural compounds. Ionic dispersants are subdivided into anionic, cationic, and amphoteric categories, contingent on their charge properties. Common anionic dispersants predominantly encompass lignin dispersants, naphthalene dispersants, humic acid dispersants, and polycarboxylic acid dispersants. Furthermore, various modified starches have been developed as dispersants. Among all types, the anionic variant exhibits the lowest production cost, hence its widespread usage [19,20].

Dispersants possess unique structural characteristics. One-dimensional dispersants encompass a linear hydrophobic end, while two-dimensional comb-like dispersants incorporate numerous hydrophilic and hydrophobic groups. Both types adhere to the coal surface via their hydrophobic ends. To enhance the adsorption capacity, a third type of dispersant, known as three-dimensional structure dispersants, has been developed. These typically comprise linear and comb structures to cater to the polar groups of coal [21,22]. The hydrophobic end and the hydrophilic end of the one-dimensional linear structure dispersant are in a straight line, such as naphthalene sulfonate formaldehyde condensate (NSF) [23,24], sodium dodecyl benzene sulfonate, and sodium dodecyl sulfate (SDS) [25], while the two-dimensional structure dispersant contains a large number of hydrophobic and polar groups, such as comb polymer sodium polystyrene sulfonate (PSS) [26]. The latter is formed by copolymerization of polymer monomer, polyethylene glycol acrylate monoester, sodium p-Phenylethane sulfonate, and acrylamide [27,28]. Preparation of these comb-like polymers involves initiation and copolymerization between diverse unsaturated monomers, which can facilitate a high coal content and low apparent viscosity via the use of super-performance one-dimensional or two-dimensional dispersants [29,30]. However, apart from the coal surface’s abundance of hydrophobic groups, it also hosts polar groups, including substances containing O, S, and N [31,32]. Therefore, the three-dimensional structure dispersant developed for this situation has more polar groups. Zhang [33] utilized renewable resources, such as tannic acid and acrylic acid, to formulate an eco-friendly polymer dispersant that possesses a jellyfish-like three-dimensional structure. Tannic acid, which comprises both hydrophobic aromatic rings and polar hydroxyl groups, closely resembles the surface properties of coal. Hence, long side chains were grafted onto the tannic acid’s plane structure, and under electrostatic repulsion and steric hindrance, aligned in parallel. This led to the formation of a hydration film on the surface of the coal particles, substantially improving the stability and reducing the viscosity of CWS. Consequently, these studies significantly reduced the production cost of dispersants, and achieved environmental sustainability during the production and use of dispersants.

Dispersants can be obtained by modifying or synthesizing natural products. Natural products such as lignin, starch, and cellulose are widely available and are indeed very low-cost and environmentally friendly. Synthetic dispersants such as naphthalene sulfonate dispersants have good performance but relatively higher costs [34]. Starch, owing to its abundance and eco-friendliness, is a frequently employed natural raw material for dispersants. However, due to its high molecular weight, starch is not chemically stable and requires degradation. This necessitates the introduction of certain groups to elevate starch’s efficacy as a dispersant, fulfilling the pulping requirements of CWS [35]. Generally, the dispersants obtained by modification are starch sulfonate, starch xanthin compound, and starch phosphate [36]. For instance, a comb-like CWS dispersant can be synthesized by polymerizing starch, acrylic acid, and styrene (SAS), which boasts numerous hydroxyl groups contributing to the hydrophilic segment [37]. These hydroxyl groups interact with water via hydrogen bonding. The hydrophobic component, the phenyl from the grafted polystyrene chains, influences the dispersant’s adsorption on coal. The molecular structure of the SAS dispersant facilitates connections between the hydrophobic groups on the coal surface and the hydrophilic groups in the water, enabling the even dispersion of coal particles in water. Natural products like starch and cellulose, which are sourced widely, are advantageous due to their low cost, renewability, eco-friendly nature, natural biodegradability, chemical stability, and biocompatibility [38,39]. Dispersants produced from these raw materials are cost-effective, cause minimal environmental pollution, and demonstrate exceptional performance. This expands the selection of dispersants, making them a prime candidate for extensive industrial application in future research [40].

In this paper, the common anionic, cationic, and non-ionic dispersants will be summarized in detail from the classification of dissociation characteristics.

**Table 1:** Dispersant types.

Basis of Classification	Subtype	Example	Characterization	Reference
Dissociation properties	Anionic	Lignin-based dispersants, naphthalene-based dispersants	Largest variety, relatively low dispersant price	[41,42]
	Cationic	Quaternary ammonium salts, heterocycles, and octadecenylamine acetate	Good dispersion	[43]
	Amphoteric	Amphoteric polycarboxylic acid dispersant	Better adsorption effect	[44]
	Non-ionic	Polyoxyethylene ether and polyoxyethylene/polyoxypropane block polyether surfactants	Easily regulated and controlled, independent of water quality and soluble matter in coal	[45]
Raw material sources	Natural product	Lignin-based dispersants, starch-based dispersants	Low-cost, abundant, and renewable	[36,46]
	Synthetic	Naphthalene sulfonate-based dispersants, polyolefin-based dispersants	Targeted modification	[34]
Dispersant structure	One-dimensional dispersants	Naphthalene sulfonic acid formaldehyde condensate (NSF), sodium salt of dodecylbenzene sulfonate, and sodium dodecyl sulfate (SDS)	With linear hydrophobic ends	[23,24,25]
	Two-dimensional dispersants	Sodium polystyrene sulfonate (PSS), polycarboxylic acid-type dispersant	Contains many hydrophilic and hydrophobic groups	[27,28]
	Three-dimensional dispersants	Dispersant TAA	Linear and comb structures	[33]

### 2.1. Anionic Dispersants

Anionic dispersants are the most researched and applied CWS dispersants at present owing to their outstanding dispersing ability, wide sources, and low price. Common anionic dispersants include lignin dispersants, humic acid-based dispersants, naphthalene-based dispersants, and polycarboxylic acid-based dispersants. The classification of anionic dispersants is shown in Table 2.

#### 2.1.1. Lignin Dispersants

Lignin dispersants are mainly obtained by modifying alkali lignin or lignosulfonate, a by-product of the paper industry. In addition to having a wide range of sources and economy, lignin dispersants can be employed to prepare coal water slurry with relatively superior stability than naphthalene-based dispersants [47]. However, the CWS viscosity prepared by such dispersants is relatively high, and the performance of lignin dispersants can be improved by modification methods such as sulfonation, polycondensation modification, and graft copolymerization modification [41,51,52].

Sulfonation modification is used to improve the hydrophilic properties of dispersants and the stability of CWS by replacing the benzene ring of lignin sulfonate molecules or the hydrogen, hydroxyl, and methoxy groups on the side chain of a benzene ring with the sulfonic group [53]. The sulfonation reaction can endow alkali lignin with good water solubility, surface activity, and reactivity. Sulfated lignin can be modified by oxidation and sulfomethylation to prepare sulfomethylated lignin [54]. Oxidation and sulfomethylation can raise the carboxyl and sulfonate contents of lignin, leading to an increase in the anion charge density. Sulfomethylated lignin can be adsorbed on coal particles and improve the fluidity of slurry more effectively.

Polycondensation modification is aimed at the phenol hydroxyl, alcohol hydroxyl, and aldehyde groups, and other unit structures of lignin sulfonate molecules that are prone to polycondensation reactions of aldehydes, phenols, lipids, and other molecules. The dispersion and adsorption of lignin dispersants can be strengthened through polycondensation modification [55]. The polycondensation reaction can endow the resulting products with certain relative molecular weights and adsorption and dispersion properties [56]. Chen et al. modified horsetail pine alkali lignin into an efficient coal water slurry dispersant, ALB, by sulfomethylation polycondensation reactions using sulfomethylated alkali lignin and sulfonated acetone formaldehyde as raw materials [41]. The experiment revealed that ALB dispersants displayed a comparatively stable viscosity reduction effect, surpassing the performance of conventional lignin dispersants. Furthermore, the molecular weight and sulfonic acid group content were identified as key factors influencing the dispersion and viscosity reduction capability of CWS. Graft copolymerization leverages the properties of lignin and its derivatives. Under the initiation process, lignin molecules can react with acrylic acid, acrylamide, styrene, hydroxyethyl methacrylate, vinyl acetate, and other functional branches, thereby enhancing their dispersion and stability. Maryam et al. [1] extracted kraft lignin and sulfonated lignin from papermaking wastewater as the source of lignin and chemical structure for acrylamide graft radical copolymerization modification initiated by thermal or redox initiators. Aliphatic hydroxyl groups were identified as the active sites of graft copolymerization, and the number of these functional groups in the lignin chain caused an important influence on the progress of graft copolymerization.

**Figure 1:**
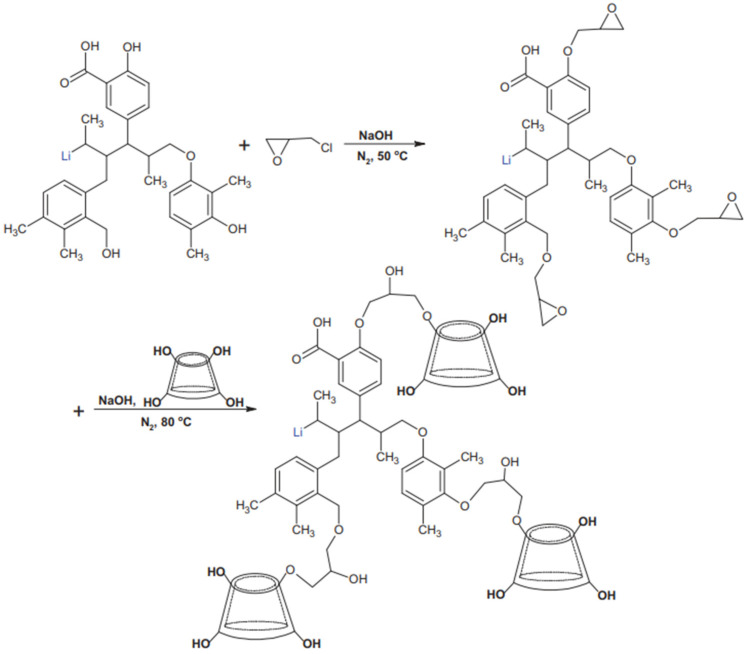
Synthetic schematic diagram of β-CD-AL.

Lu et al. [57] copolymerized β-cyclodextrin (β-CD) and chlorotrione epoxide into alkali lignin to synthesize a modified alkali lignin dispersant (β-CD-AL), which dramatically increased the stability of the lignin dispersant pulping and reduced the viscosity of the slurry in their study. The synthesis scheme of the dispersant is described in Figure 1. The effects of the β-CD content on the dispersibility, zeta potential, and adsorption properties of β-CD-AL were also investigated. It was found that the stability was gradually enhanced with the increase in the copolymerized β-CD dosage under the synergistic effect of electrostatic repulsion and spatial site resistance. In addition, the amount of copolymerized β-CD reached a peak value for the optimal viscosity reduction effect.

#### 2.1.2. Humic Acid-Based Dispersants

Low-rank coal, such as lignite, contains a large amount of humic acid. Humic acid dispersants, extracted from low-rank coal such as lignite, present good dispersion performance and can be used alone [58]. It has been confirmed that the lower the maturity of raw coal, the better the viscosity reduction effect of the prepared dispersants for CWS. However, the disadvantages are that these dispersants are sensitive to metal ions, propensity form precipitates, lead to prepared slurry with poor stability, and propose correspondingly higher requirements for pulping water quality [42,48].

Humic acid is rich in condensed aromatic units, which are similar to the structure of coal, and thus can be tightly adsorbed on the coal surface [59,60]. In addition, humic acid encompasses active groups, such as hydroxyl and carboxyl groups, providing the possibility for chemical modification of humic acid [61]. Currently, studies on the modification of humic acid as a dispersant focus on the sulfonation, nitration, sulfomethylation, and graft copolymerization of humic acid molecules. The purpose is to introduce functional groups with strong hydrophilicity into humic acid molecules [62,63]. Zhang et al. [48] fabricated a novel humic acid-based dispersant, humic acid-grafted sodium polystyrene sulfonate (HA-g-pssNa). This dispersant possesses a hydrophobic humic acid core and a hydrophilic sodium polystyrene sulfonate side chain, synthesized through a surface acylation reaction and atom transfer radical polymerization of humic acid. Various properties of HA-g-pssNa, pssNa, and naphthalene sulfonate formaldehyde condensate (NSF) as dispersants for the preparation of CWS were compared. The results revealed that the pulping properties of HA-g-pssNa were reinforced with the increase in pssNa side chain length. In the case of the appropriate chain length of HA-g-pssNa, the CWS prepared with this dispersant achieved good apparent viscosity and static stability, with superior performance than pssNa and NSF.

Kang et al. [42] modified sodium humate solution by sulfomethylation to prepare sulfomethylated humic acid dispersant (LSHA dispersant) and determined the optimal process conditions for modification through orthogonal experiments. Compared with the slurry-forming performance of commercial sodium naphthalenesulfonate dispersants, both of them can meet the requirements of CWS gasification, but the LSHA dispersant is better in terms of stability.

**Figure 2:**
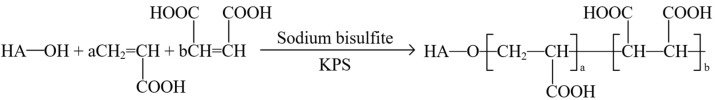
Humic acid-based dispersant polymerization reaction scheme.

A humic acid-based polycarboxylate dispersant for CWS can be synthesized by copolymerizing humic acid, acrylic acid, and maleic acid, and its dispersion performance is much better than that of humic acid before copolymerization modification. The synthesis scheme of the dispersant is described in Figure 2 [64]. When the dosage of the dispersant reached 0.5 wt%, the apparent viscosity of the CWS was 505 mPa·s, while the permeability reached 85.45% after 96 h. The stability of the CWS was 12.87% higher than that of CWS prepared directly with humic acid as a dispersant. And the maximum concentration of the coal water slurry could reach up to 70 wt%. Overall, the aforementioned studies have greatly broadened the application scope of humic acid dispersants.

#### 2.1.3. Naphthalene-Based Dispersants

**Figure 3:**
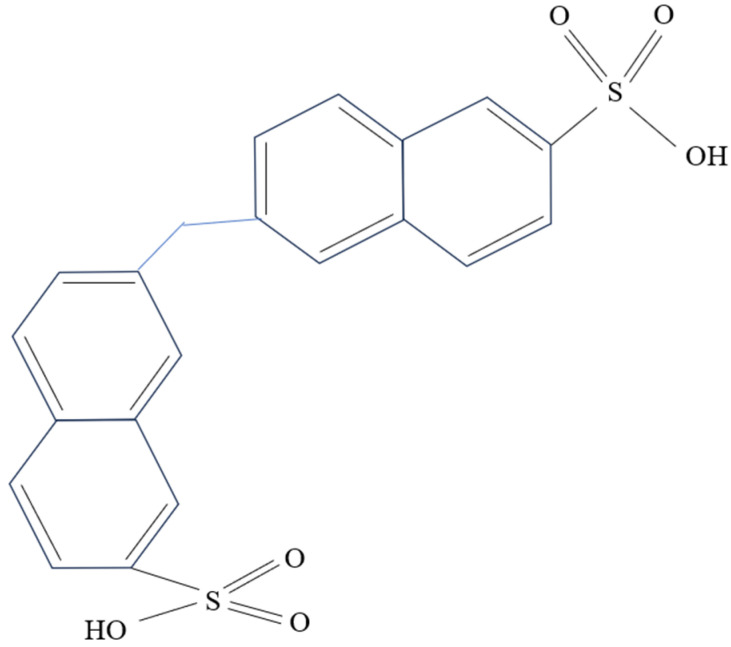
Structural formula of sodium methylene naphthalene sulfonate.

Naphthalene-based dispersants, primarily comprised of naphthalene sulfonic acid polymers, are the most prevalent dispersants on the market. The structure of typical naphthalene dispersants is depicted in Figure 3. These dispersants offer superior dispersion performance, viscosity reduction, and slurry fluidity when compared with lignin-based dispersants. However, they also have notable disadvantages including high cost, suboptimal slurry stability, and a propensity for precipitation [47,49].

The dispersion performance of naphthalene-based dispersants can primarily be adjusted by varying the degree of condensation and sulfonation [65]. An increased degree of condensation correlates positively with the binding strength and slurry effect of coal. However, for coal molecules with medium and low degrees of metamorphism, there exists a significant steric hindrance effect. This results in a weakened bond between the dispersant and the coal, despite an increase in the molecular chain length of the dispersants concurrent with the condensation degree. This suggests an optimal value for the coagulability of coal water slurries derived from medium and low-grade coals exists [66,67]. Another strategy for improving the performance of naphthalene dispersants is graft modification. Modifying the branch chain length through graft copolymerization can produce modified naphthalene dispersants with varying chain lengths. This can be achieved by adjusting the ratio of ethylene oxide to aromatic monomer.

Currently, naphthalene sulfonate formaldehyde condensate (NSFC) has emerged as a widely used dispersant in CWS applications due to its effective viscosity reduction properties. At present, scholars mainly study the effects of the reaction conditions, sulfonation quality, and degree of polymerization on its dispersion performance [34]. Upon the addition of NSFCs to CWS, the coal surface’s hydrophobicity decreases while the conversion of weakly bound water to free water is facilitated, thereby enhancing water fluidity. Consequently, the hydrophilicity of the coal surface is increased and the viscosity is significantly reduced [68]. Due to its cost-effectiveness and superior viscosity control characteristics, NSFC holds potential for broader industrial applications compared to other dispersants [69].

#### 2.1.4. Polycarboxylic Acid Dispersants

**Figure 4:**
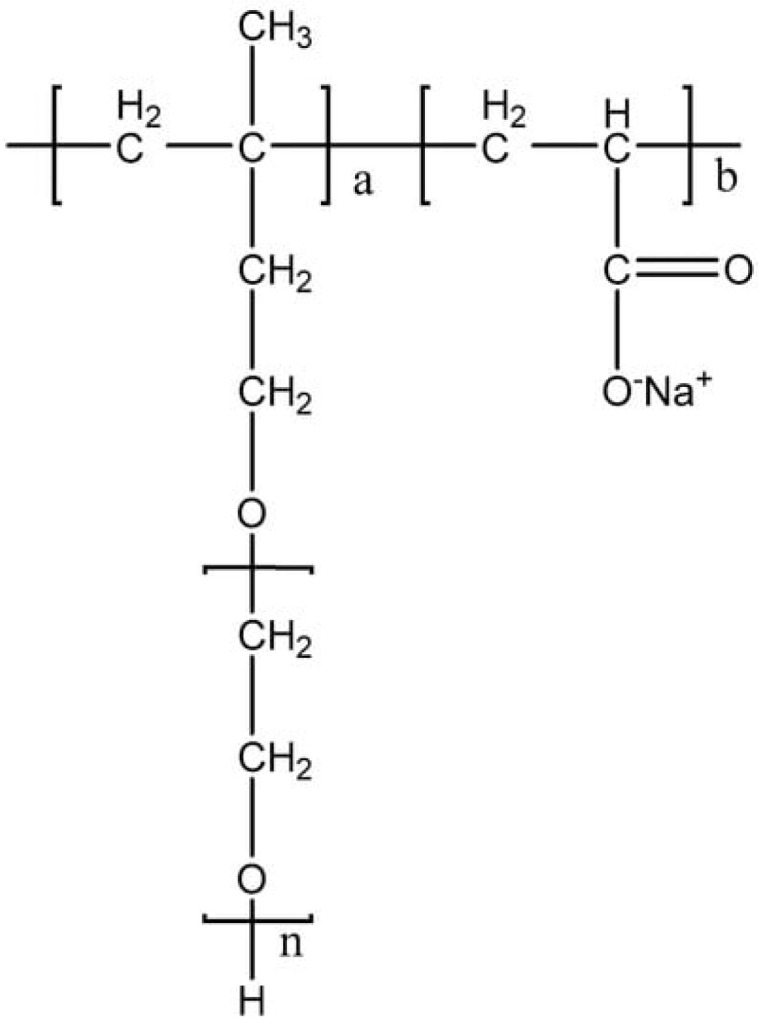
Structure of polycarboxylate dispersant.

The molecular structure of polycarboxylate-based additives consists of comb-shaped surfactants with graft copolymers. The main chain is polymerized by active monomers comprising functional groups, and the side chain is grafted onto active monomers containing functional groups and the main chain [27,50]. The structure is easy to design and can be modified according to different needs. Therefore, the viscosity reduction effect of polycarboxylate-based dispersants is stronger than that of traditional lignin dispersants and naphthalene dispersants. Combined with the advantage of low pollution, such dispersants enjoy a wider range of applications [70,71]. The structure of common polycarboxylate dispersant is shown in Figure 4 [72].

Polycarboxylate dispersants are easy to prepare because they can regulate the adsorption capacity on coal surfaces and improve the chemical properties of coal surfaces by introducing polycarboxylate. Zhu [70] and others adopted ammonium persulfate sodium bisulfite as a redox catalyst to synthesize a new amphoteric polycarboxylate dispersant for CWS by using sodium p-phenylethylsulfonate, polyethylene glycol acrylate monoester, and ethyl trimethyl ammonium methacrylate. When the dosage of the dispersant was 0.3 wt%, the maximum concentration of CWS could reach 65.0 wt%. Polycarboxylate dispersants containing anionic and cationic groups lead to a better anchoring effect on coal through ion adsorption.

In recent years, it is a research hotspot to adjust the synthesis scheme of polycarboxylate dispersants to obtain better-performing dispersants, such as controlling the ratio of acrylic acid to sodium phenylene sulfonate, initiator composition, and temperature. The most suitable PC dispersants for pulping were selected according to the viscosity of each type of CWS at 100 s^−1^, shear thinning behavior, static stability (>14), and maximum solid loads (>55 wt%). Polycarboxylate dispersants are effectively adsorbed on the surface of coal particles through horizontal multipoint adsorption, mainly through the interaction between the hydrophobic groups of dispersant molecules and the hydrophobic regions of coal particles, finally intensifying the hydrophilicity of coal particles and the stability of the hydration membrane [73].

### 2.2. Cationic Dispersants

**Figure 5:**
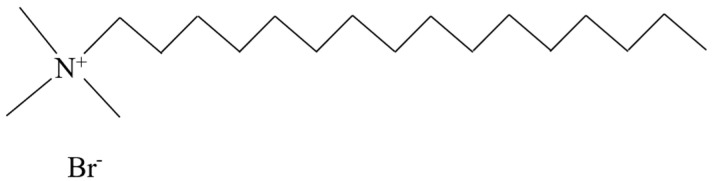
Structure diagram of cetyltrimethylammonium bromide.

The molecular structure of cationic dispersants typically comprises two components: positively charged non-polar hydrophilic groups and lipophilic hydrocarbon chains. Unlike anionic types, cationic dispersants facilitate the dispersion of particles in water into a colloidal solution via interaction between the positively charged molecular groups and the negatively charged particle surface. Currently, quaternary ammonium salts, heterocycles, and octadecenylamine acetate represent some of the widely used cationic dispersants on the market [43]. The classification of cationic dispersants is shown in Table 3. Figure 5 presents a typical molecular structure diagram of a quaternary ammonium salt dispersant.

Nurcan Acet investigated the impact of the cationic dispersant polyethyleneimine (PEI) on the dispersion performance of TiC nanoparticles in electrolytes [74]. Experimental results indicated that PEI at a concentration of 125 ppm optimized the dispersion of TiC nanoparticles in electrolytes, allowing particles to be uniformly distributed within the deposition layer without affecting the kinetics of electrodeposition. Without a dispersant, the TiC concentration in the coating remained largely unaffected by current density. However, in a bath containing PEI, an increase in current density corresponded to a reduction in TiC concentration.

While cationic dispersants offer excellent dispersion effects, they should not be combined with anionic dispersants to prevent reaction with the carboxyl in materials. Consequently, some researchers have explored the combination of cationic dispersants with non-ionic dispersants. Routray et al. [75] examined the interaction between the non-ionic dispersant saponin and the cationic surfactant di-dialkyl ammonium bromide (DDAB) in high-concentration CWS containing four types of coal samples with bimodal particle size distribution. The observed surface tension was used to explain the dispersion stability of the mixed additive system and to optimize the ratio of the two dispersants. Both saponin and DDAB formed robust water dispersion bonds tightly adhering to the coal surface, resulting in a highly stable CWS system.

### 2.3. Non-Ionic Dispersants

**Figure 6:**
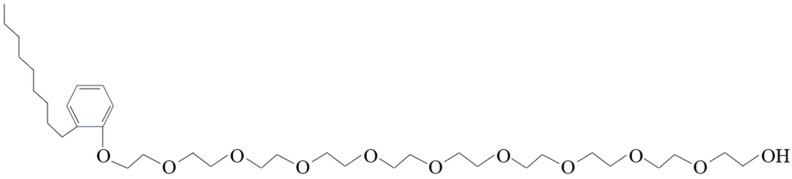
Molecular structure of alkylphenol polyoxyethylene ether.

The primary types of non-ionic CWS dispersants are polyoxyethylene ether and polyoxyethylene block polyether surfactants, with the former attracting more attention. Characterized by their non-ionization in water, non-ionic dispersants can control both hydrophilic and hydrophobic groups, making them less susceptible to the influence of water quality and the substances in coal on the dispersion effect compared to other dispersants. At the same time, there is no need for a stabilizer when using non-ionic dispersants. They are also the most expensive dispersants for CWS [76]. Several common non-ionic dispersants are shown in Table 4. A CWS dispersant prepared by non-ionic surfactants extracted from natural plants is a relatively low-cost, environment-friendly, and strong non-ionic dispersant. Figure 6 illustrates the structure of a typical non-ionic dispersant.

The minimum apparent viscosity that CWS can achieve with polyoxyethylene ether as a dispersant is closely related to the polyoxyethylene adduct number [45]. Alkyl polyoxyethylene ethers with more alkyl carbon atoms have an optimal addition number. Li [77] utilized two non-ionic dispersants, polyoxyethylene dodecyl phenol ether (PDPE) and polyoxyethylene lauryl ether (PLE), as CWS dispersants and compared their slurry forming performance. Both dispersants contained 30% ethylene oxide. At a CWS viscosity of 1000 mPa·s, the maximum concentrations of PDPE and PLE were 67.60% and 62.95%, respectively. PDPE displayed easier adsorption onto coal than PLE and formed more stable bonds due to the P-P stacking effect, leading to more uniform coal dispersion in the solution.

Lauryl alcohol polyoxyethylene ether, with the molecular formula (C_2_H_4_O) _n_·C_12_H_26_O, is a non-ionic copolymer exhibiting good water solubility, resistance to acid and alkali, hard water resistance, and high stability. Zhao [78] analyzed the influence of dispersant mass fraction and CWS mass fraction on the ultimate settlement concentration, and concluded that the maximum pulping mass fraction reached 72% when the dispersant mass fraction was 1.0%, which was less than the ultimate settlement concentration. A dispersant mass fraction of 1.0% yielded the maximum and minimum values for the ultimate settlement concentration and apparent viscosity, respectively.

**Table 4:** Non-ionic dispersant types.

Category	Type	Characteristic	Reference
Non-ionic dispersant	Polyoxyethylene ether	Good wettability, permeability, and dispersion	[76]
	Polyoxyethylene block polyether	High chemical stability	[79]
	Polyoxyethylene alkyl alcohol amide	Good stability and hydrolysis resistance	[78]

## 3. Mechanism of CWS Dispersants

**Figure 7:**
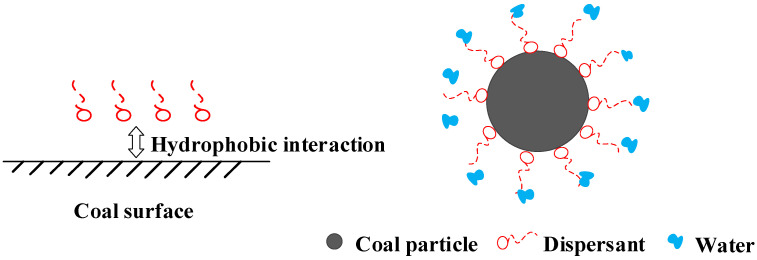
Adsorption mechanism of dispersant.

The effectiveness of dispersants correlates strongly with the chemical characteristics of the coal particle surface [36]. Besides numerous hydrophobic groups, the coal surface also contains several polar groups, comprising O, S, and N elements. Figure 7 illustrates that the dispersant is adsorbed onto the coal surface through charge adsorption or functional groups, thereby affecting the steric resistance, electrostatic interaction, and hydrophilicity between particles. This, in turn, alters the rheological properties of the slurry [80]. Consequently, the impact of dispersants on the chemical properties of coal surfaces has become a focal point of research. Currently, studies on the viscosity reduction mechanism of dispersants primarily focus on three aspects: the wetting dispersion effect, the electrostatic repulsion effect, and the steric hindrance effect [71,81,82].

### 3.1. Wetting and Dispersing

In the process of preparing CWS, coal particles are thoroughly mixed with water, transitioning the gas–solid interface into a solid–liquid one. Hence, a fundamental requirement for CWS preparation is the complete wetting of coal particles in water. Given that coal is hydrophobic and CWS dispersants possess an amphiphilic structure [46,75], these dispersants, when adsorbed onto the coal surface, can interact with water molecules to fully saturate the coal particles.

**Figure 8:**
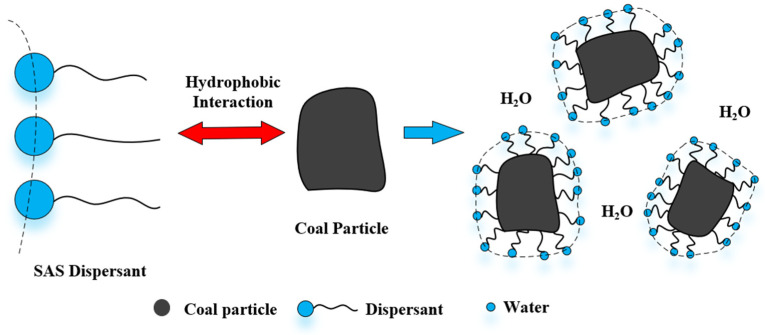
Wetting mechanism of dispersant.

Figure 8 illustrates that the hydrophobic end of the SAS dispersant binds to the hydrophobic groups on the coal particle surface, while the hydrophilic end extends into the water, establishing a directional arrangement. This alters the coal particle surface into a hydrophilic one, promoting the adsorption of numerous water molecules to form a hydration film, ensuring complete wetting [30,83]. Evidence for this can be seen in the changes in the coal particle contact angle before and after dispersant use [84]. Moreover, dispersants alter the surface tension of water, further boosting the wetting and dispersion of coal in water [33]. The hydrophobicity of the coal surface and its spontaneous aggregation in an aqueous solution are linked to its wettability. The lower the surface tension of the aqueous solution, the easier it is for the coal particles to be wetted [85].

### 3.2. Electrostatic Repulsion

Based on the DLVO theory, for the stable existence of colloidal particles, the electrostatic repulsion between particles must exceed the van der Waals attractive forces [86]. Therefore, when ionic dispersants are adsorbed on the surface of coal particles, the electrostatic repulsion between the coal particles is generated due to carrying the same charge. When this repulsion force is greater than the van der Waals force between coal particles, the coal water slurry system can maintain relative stability. Based on the extended DLVO theory, the van der Waals interaction energy (*E_v_*), electrostatic interaction energy (*E_e_*), hydrophobic interaction energy (*E_h_*), and spatial stability energy (*E_S_*) are compared. The total interaction energy (*E_T_*) is calculated as follows [87]:

(1) $$E_{T}=E_{v}+E_{e}+E_{h}+E_{s}\;$$

In the calculation process, it is assumed that all solid particles are spheres with a characteristic average size. The two spherical particles of van der Waals interaction energy (*E_v_*) are represented by the following equations [88]:

(2) $$E_{v}=-\frac{A_{132}}{6H}\frac{R_{1}R_{2}}{R_{1}+R_{2}}$$

(3) $$A_{132}=(\sqrt[{}]{A_{11}}-\sqrt[{}]{A_{33}})(\sqrt[{}]{A_{22}}-\sqrt[{}]{A_{33}})$$

*R_1_* and *R_2_* represent the average radii of particle 1 and particle 2. *H* is the distance between particles (nm). *A_132_* refers to the Hamaker constant of particle 1 and particle 2 involving in medium 3.

Two spherical particles with electrostatic interaction energy (*Ee*) can be calculated by the Formula (4) [89]:

(4) $$E_{e}=\frac{\varepsilon \varepsilon_{0}\pi R_{1}R_{2}}{R_{1}+R_{2}}\left[2\varphi_{1}\varphi_{2}\ln\frac{1+e^{-kh}}{1-e^{-kh}}+(\varphi_{1}^{2}+\varphi_{2}^{2})\ln\left(1-e^{-2kh}\right)\right]$$

ε denotes the relative dielectric constant of water, with a value of 78.5 C^2^·m/J. $\varepsilon_{0}$ is the dielectric constant of vacuum [90], with its value of 8.854 × 10^−12^ C^2^·m/J. $\varphi_{1}$ and $\varphi_{2}$ represent the surface potential of two particles respectively; *K^−1^* is the Debye length [91], about 0.104 nm.

The hydrophobic interaction energy (*E_h_*) can be determined by Formula (5) [92]:

(5) $$E_{h}=-exp(a\frac{\cos\theta_{1}+\cos\theta_{2}}{2}+b)\frac{R_{1}+R_{1}}{(R_{1}+R_{2})H^{2}}$$

$\theta_{1}$ and $\theta_{2}$ are the corresponding contact angles of the two particles. The optimal values of parameters *a* and *b* are −7.0 and −18.0, respectively [93].

The spatial potential resistance stabilization energy (*E_s_*) of two spherical particles can be calculated as follows [87]:

(6) $$V_{s}=\frac{16\pi {\left(\frac{R_{1}R_{2}}{R_{1}+R_{2}}\right)}^{2}(\frac{2\delta_{1}\delta_{2}}{\delta_{1}+\delta_{2}}-0.5H)}{A_{P}(R+\frac{2\delta_{1}\delta_{2}}{\delta_{1}+\delta_{2}})}kT\ln\left(\frac{\frac{4\delta_{1}\delta_{2}}{\delta_{1}+\delta_{2}}}{H}\right)$$

(7) $$A_{P}={2\sqrt[{}]{3}(\frac{M}{4\sqrt[{}]{2}N_{A}\rho })}^{\frac{2}{3}}$$

where *k* indicates the Boltzmann constant with a value of 1.381 × 10^−23^ J/K; T means the experimental temperature, with its value of 298 K; *A_P_* is the area of dispersant absorbed on the particle surface (m^2^); M is the relative molecular mass of dispersant molecules; *ρ* refers to the density of dispersant molecules; and δ_1_ and δ_2_ are the thickness of the adsorbed layers of the two particles.

**Figure 9:**
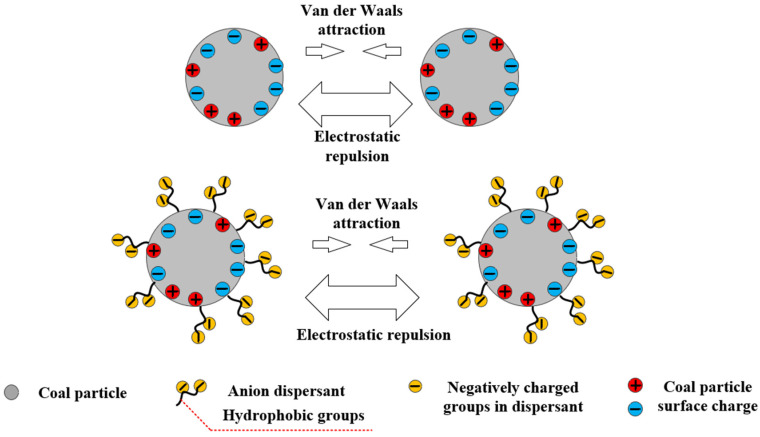
Electrostatic repulsion mechanism of dispersant.

The addition of ionic CWS dispersants in CWS can change the wetting degree and potential of coal. When the coal particles are adsorbed with anionic dispersants, the coal particles are dispersed due to the increase in electrostatic repulsion on the surface, which makes the CWS system stable. As shown in Figure 9, the hydrophobic group of the main chain of anionic dispersants is bonded to the coal surface, which plays an anchoring role in improving the adsorption force between anionic dispersants and coal particles. The hydrophilic group of the branched chain in dispersants stretches from the coal surface to the solution and changes the wettability and surface potential of the coal particle surface, achieving the effects of dispersion and viscosity reduction [94,95].

However, the presence of soluble minerals in both industrial water and coal introduces significant amounts of high-valence metal ions, such as Mg^2+^ and Ca^2+^, into the CWS system. These metal ions reduce the coal surface potential, which remains largely unaffected by the addition of dispersants. This suggests that the enhancement of electrostatic repulsion between coal particles is not the sole determining factor contributing to particle dispersion, and the synergistic effect of other factors must also be considered [11,14]. In a study by Chen et al. [23] on the dispersion mechanism of naphthalene sulfonate formaldehyde (NSF) in CWS, it was discovered that the apparent viscosity and yield stress of CWS significantly decreased with the addition of 1.5% NSF, aligning with the saturated adsorption value of NSF on superfine coal. Furthermore, the zeta potential and contact angle of finely pulverized coal decreased with increasing NSF dosage, indicating that the coal surface experienced greater electrostatic repulsion force and improved wetting ability after NSF addition. This observation supports the notion that electrostatic repulsion alone is not the sole factor influencing coal particle dispersion in CWS.

### 3.3. Spatial Steric Effects

**Figure 10:**
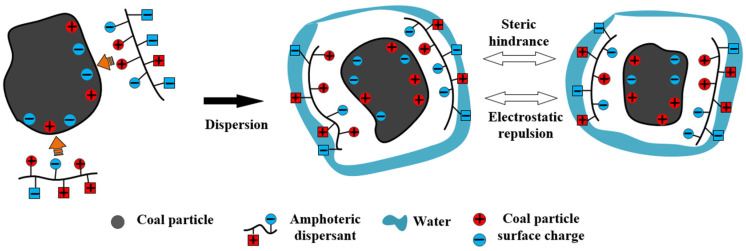
Mechanism of action of the novel dispersant P (SS-co-AA-co-DMDAAC).

The formation of an adsorption layer on the surface of coal particles creates a barrier that hinders particle aggregation in the CWS system. This spatial barrier effect prevents the particles from approaching each other, making aggregation difficult. Entropy repulsive dispersion refers to the phenomenon where particles with an adsorption layer experience a decrease in the freedom of polymer dispersants within the layer, resulting in a decrease in the entropy of the adsorbed molecules. The system then spontaneously tends to increase entropy, causing the particles to separate again [96,97]. Using the mechanism of action of a novel amphoteric copolymer dispersant, P (SS-co-AA-co-DMDAAC), as an example (refer to Figure 10), the dispersant is adsorbed onto the coal surface through the attraction of heterogeneous charges and hydrogen bonding. The dispersion effect is achieved through the formation of a surface water adsorption film and electrostatic repulsion [44]. The adsorption of the dispersant onto the coal surface primarily relies on spatial steric effects, supplemented by electrostatic repulsion, working together to enhance the performance of the CWS.

## 4. Conclusions and Prospects

Given the rapid advancement and extensive application of coal water slurry (CWS) dispersants, this paper assimilates information on the classification and mechanism of these dispersants to facilitate a comprehensive understanding. To elucidate the characteristics of CWS dispersants, we propose a classification based on dissociation properties, product source, and structure, in addition to summarizing the adsorption and action mechanisms. Despite their widespread use, CWS dispersants present many challenges that necessitate resolution. Consequently, we propose future research directions: (1) The suitability of dispersants is relatively exclusive, making the development of dispersants apt for a range of coal samples often costly. To address multitasking demands and cost-effectiveness, compound dispersant development is a targeted research direction. (2) Many dispersants exhibit toxicity, hence the increasing demand for eco-friendly options. (3) While some novel CWS types demonstrate impressive performance, the associated costs are relatively high. Consequently, the development of low-cost and high-performance CWS is a vital research focus. For instance, the utilization of industrial waste and low-cost natural products like engine oil, natural resin, and starch in dispersant preparation is an area of active research. Future trends in CWS dispersant research are likely to emphasize cost reduction, performance enhancement, and environmental sustainability.

## Figures and Tables

**Table 2:** Anionic dispersant types.

Category	Type	Characteristic	Reference
Anionic dispersant	Lignin dispersant	Low cost and good stability	[47]
	Humic acid-based dispersants	Good viscosity reduction effect and poor stability	[42,48]
	Naphthalene-based dispersants	Good liquidity and poor stability	[47,49]
	Polycarboxylic acid dispersants	Good performance, low pollution	[27,50]

**Table 3:** Cationic dispersant types.

Category	Type	Characteristic	Reference
Cationic dispersant	quaternary ammonium salts	Good stability	[43]
	heterocycles	Heterocyclic structure	[74]
	octadecenylamine acetate	Strong adsorption capacity	[75]

## Data Availability

No new data were created or analyzed in this study. Data sharing is not applicable to this article.
